# Does the DNA barcoding gap exist? – a case study in blue butterflies (Lepidoptera: Lycaenidae)

**DOI:** 10.1186/1742-9994-4-8

**Published:** 2007-03-07

**Authors:** Martin Wiemers, Konrad Fiedler

**Affiliations:** 1 Department of Population Ecology, Faculty of Life Sciences, University of Vienna, Althanstrasse 14, 1090 Vienna, Austria

## Abstract

**Background:**

DNA barcoding, i.e. the use of a 648 bp section of the mitochondrial gene cytochrome *c *oxidase I, has recently been promoted as useful for the rapid identification and discovery of species. Its success is dependent either on the strength of the claim that interspecific variation exceeds intraspecific variation by one order of magnitude, thus establishing a "barcoding gap", or on the reciprocal monophyly of species.

**Results:**

We present an analysis of intra- and interspecific variation in the butterfly family Lycaenidae which includes a well-sampled clade (genus *Agrodiaetus*) with a peculiar characteristic: most of its members are karyologically differentiated from each other which facilitates the recognition of species as reproductively isolated units even in allopatric populations. The analysis shows that there is an 18% overlap in the range of intra- and interspecific *COI *sequence divergence due to low interspecific divergence between many closely related species. In a Neighbour-Joining tree profile approach which does not depend on a barcoding gap, but on comprehensive sampling of taxa and the reciprocal monophyly of species, at least 16% of specimens with conspecific sequences in the profile were misidentified. This is due to paraphyly or polyphyly of conspecific DNA sequences probably caused by incomplete lineage sorting.

**Conclusion:**

Our results indicate that the "barcoding gap" is an artifact of insufficient sampling across taxa. Although DNA barcodes can help to identify and distinguish species, we advocate using them in combination with other data, since otherwise there would be a high probability that sequences are misidentified. Although high differences in DNA sequences can help to identify cryptic species, a high percentage of well-differentiated species has similar or even identical *COI *sequences and would be overlooked in an isolated DNA barcoding approach.

## Background

Molecular tools have provided a plethora of new opportunities to study questions in evolutionary biology (e.g. speciation processes) and in phylogenetic systematics. Only recently, however, have claims been made that the sequencing of a small (648 bp) fragment at the 5' end of the gene cytochrome *c *oxidase subunit 1 (*COI *or cox1) from the mitochondrial genome would be sufficient in most Metazoa to identify them to the species level [1,2]. This approach called "DNA barcoding" has gained momentum and the "Consortium for the Bar Code of Life (CBOL)" founded in September 2004 intends to create a global biodiversity barcode database in order to facilitate automated species identifications. Right from the start, however, this approach received opposition, especially from the taxonomists' community [3-8]. Some arguments in this debate are political in nature, others have a scientific basis. Concerning the latter, one of the most essential arguments focuses on the so-called "barcoding gap". Advocates of barcoding claim that interspecific genetic variation exceeds intraspecific variation to such an extent that a clear gap exists which enables the assignment of unidentified individuals to their species with a negligible error rate [1,9,10]. The errors are attributed to a small number of incipient species pairs with incomplete lineage sorting (e.g. [11]). As a consequence, establishing the degree of sequence divergence between two samples above a given threshold (proposed to be at least 10 times greater than within species [10]) would indicate specific distinctness, whereas divergence below such a threshold would indicate taxonomic identity among the samples. Furthermore, the existence of a barcoding gap would even enable the identification of previously undescribed species ([11-13] but see [14]). Possible errors of this approach include false positives and false negatives. False positives occur if populations within one species are genetically quite distinct, e.g. in distant populations with limited gene flow or in allopatric populations with interrupted gene flow. In the latter case it must be noted that, depending on the amount of morphological differentiation and the species concept to be applied, such populations may also qualify as 'cryptic species' in the view of some scientists. False negatives, in contrast, occur when little or no sequence variation in the barcoding fragment is found between different biospecies (= reproductively isolated population groups sensu Mayr [15]). Hence, false negatives are more critical for the barcoding approach, because the existence of such cases would reveal examples where the barcoding approach is less powerful than the use of other and more holistic approaches to delimit species boundaries.

Initial studies on birds [10] and arthropods [9,16] appeared to corroborate the existence of a distinct barcoding gap, but two recent studies on gastropods [17] and flies [18] challenge its existence. The reasons for these discrepancies are not entirely clear. Although levels of *COI *sequence divergence differ between higher taxa (e.g. an exceptionally low mean *COI *sequence divergence of only 1.0% was found in congeneric species pairs of Cnidaria compared to 9.6–15.7% in other animal phyla [2]), Mollusca (with 11.1% mean sequence divergence between species) and Diptera (9.3%) are not peculiar in this respect. Meyer & Paulay [17] assume that insufficient sampling on both the interspecific and intraspecific level create the artifact of a barcode gap. Proponents of barcoding might argue, however, that the main reason for this overlap is the poor taxonomy of these groups, e.g. cryptic species may have been overlooked which are differentiated genetically but very similar or even identical in morphology.

If the barcode gap does not exist, then the threshold approach in barcoding becomes inapplicable. Although more sophisticated techniques (e.g. using coalescence theory and statistical population genetic methods [19-21]) can sometimes help to delimit species with overlapping genetic divergences, these approaches require additional assumptions (e.g. about the choice of population genetic models or clustering algorithms) and are only feasible in well-sampled clades.

Barcoding holds promise nonetheless especially in the identification of arthropods, the most species-rich animal phylum in terrestrial ecosystems. Identification of arthropods is often extremely time-consuming and generally requires taxonomic specialists for any given group. Moreover, the fraction of undescribed species is particularly high, as opposed to vertebrates. Hence, there is substantial demand for improved (and rapid) identification tools by scientists who seek identification of large arthropod samples from complex faunas. Therefore arthropods deserve to be considered the yard-stick for the usefulness of barcoding approaches among Metazoa and it is not surprising that several recent studies have tried to apply DNA barcoding in arthropods [9,11-13,16,18,19,22-27]. Diversity is concentrated in tropical ecosystems, but measuring intra- and interspecific sequence divergence in tropical insects is hampered by the fragmentary knowledge of most taxa. In contrast, insects of temperate zones, and most notably the butterflies of the Holarctic region, are well known taxonomically compared to other insects. The species-rich Palaearctic genus (or subgenus) *Agrodiaetus *provides an excellent example to test the existence of the barcode gap in arthropods. This genus is exceptional because of its extraordinary interspecific variation in chromosome numbers which have been investigated for most of its ca 120 species ([28-30] and references therein). As a result several cryptic species which hardly or not at all differ in phenotype have been discovered (e.g. [31-39]). Available evidence suggests that apart from a few exceptions (e.g. due to supernumerary chromosomes) differences in chromosome numbers between butterfly species are linked to infertility in interspecific hybrids [40]. This is due to problems in the pairing of homologous chromosomes during meiosis. Since major differences in chromosome numbers are indicative of clear species boundaries, they are helpful also to infer species-level differentiation for allopatric populations. *Agrodiaetus *butterflies therefore are an ideal case for testing the validity of the barcoding approach. If valid, then it must be possible to safely recognize all species that can be distinguished by phenotype, karyotype or both character sets with reference to sequence divergences alone. On the contrary, failure of DNA barcodes to differentiate between species that are distinguished by clear independent evidence would undermine the superiority of the barcoding approach, which has especially been attributed to taxa with "difficult" classical taxonomy, such as *Agrodiaetus*.

## Results

### Intraspecific divergence

The average divergence in 1189 intraspecific comparisons is 1.02% (SE = 1.13%). 95% of intraspecific comparisons have divergences of 0–3.2%. The few values higher than 3.2% are conspicuous and probably due to misidentifications (*Lampides boeticus, Neozephyrus japonicus, Arhopala atosia, Agrodiaetus kendevani*, see below), unrecognized cryptic species (*Agrodiaetus altivagans *[41], *Agrodiaetus demavendi *[30]), hybridization events (*Meleageria marcida *[30,42]) or any of those (*Agrodiaetus mithridates, Agrodiaetus merhaba*).

The evidence for the possible misidentifications is the following:

• *Lampides boeticus *is the most widespread species of Lycaenidae and a well-known migrant which occurs throughout the Old World tropics and subtropics from Africa and Eurasia to Australia and Hawaii. Apart from a single unpublished sequence (AB192475), all other *COI *GenBank sequences of this species (from Morocco, Spain and Turkey) are identical with each other or only differ in a single nucleotide (= 0.15% divergence). They are also nearly identical to two specimens of *Lampides boeticus *in the CBOL database (BOLD) [43] from Tanzania and another sequence of this species from Papua New Guinea (Wiemers, unpubl. data). The GenBank sequence AB192475 (of unknown origin, but possibly from Japan), however, differs strongly (8.2–8.7%) from all other *Lampides boeticus *sequences and therefore we assume this to represent a distinct species. Its identity however remains a mystery because it is not particularly close to any other GenBank sequence and a request for a check of the voucher specimen has remained unanswered for more than a year.

• The questionable unpublished sequence of *Neozephyrus quercus *(AB192476) is identical to a sequence of *Favonius orientalis *and therefore probably represents this latter species which is very similar in phenotype but well differentiated genetically (4.8% divergence).

• A similar situation constitutes the questionable unpublished sequence of *Arhopala atosia *(AY236002) which is very similar (0.4%) to a sequence of *Arhopala epimuta*.

• *Agrodiaetus kendevani *is a local endemic of the Elburs Mts. in Iran. The two sequences of this species in the NCBI database which exhibit a divergence of 5.4% have been published in two different papers by the same work group [29,44]. While one of them is identical to a sequence of *Agrodiaetus pseudoxerxes*, the other one is nearly identical to *Agrodiaetus elbursicus *(0.2% divergence). These latter two species however belong to separate species groups [30] and thus conspecificity of the two sequences of *A. kendevani *is very improbable as there is no evidence of hybridization between members of different species groups in *Agrodiaetus *[30].

Higher intraspecific divergence values are also found between North African and Eurasian populations of *Polyommatus amandus *(3.8%) and *Polyommatus icarus *(5.7–6.8%). In the former species the North African population is also well differentiated in phenotype (ssp. *abdelaziz*), while in the latter species phenotypic differences have never been noted. Cases with substantial, but lower genetic divergence between North African and European populations which do not correspond to differentiation in phenotype also occur in the butterflies *Iphiclides (podalirius) feisthamelii *(2.1%; [30]) and *Pararge aegeria *(1.9%; [45]). In all cases these allopatric populations may actually represent distinct species, although we do not currently have additional evidence in support of this hypothesis.

Although some of the other higher divergence values >2% are possibly due to cryptic species (e.g. in *Agrodiaetus demavendi*) or hybridization between closely related species (e.g. in the species pair *Lysandra corydonius *and *L. ossmar*, as evidenced by the comparative analysis of the nuclear rDNA internal transcribed spacer region *ITS-2 *[30]), most of those values represent cases in which there is hardly any doubt regarding the conspecificity of samples. The highest such value is 2.9% between distant populations of the widespread *Agrodiaetus damon *(from Spain and Russia). Outside the genus *Agrodiaetus *high values are also found between North African and Iranian populations of *Lycaena alciphron *(2.7%), Spanish and Anatolian populations of *Polyommatus dorylas *(2.3%) and even between Polish and Slovakian populations of *Maculinea nausithous *(2.3%). Table 1 lists mean intraspecific divergences in those species that are represented by more than one individual in the data set.

**Table 1:** Intraspecific nucleotide divergences

**Species**	**No. of individuals**	**Mean percent divergence**	**Standard error (%)**	**Range (%)**	**Monophyly**	**corrected**
*Acrodipsas aurata*	3	0.2	0.1	0.2 – 0.3	Mono	Mono
*Acrodipsas brisbanensis*	8	1.0	0.5	0.2 – 1.6	Mono	Mono
*Acrodipsas cuprea*	6	0.5	0.3	0.2 – 0.9	Mono	Mono
*Acrodipsas hirtipes*	2	1.0		---	Mono	Mono
*Acrodipsas mortoni*	2	0.2		---	Mono	Mono
*Agrodiaetus admetus*	4	1.7	0.7	0.5 – 2.5	Poly	Poly
*Agrodiaetus ainsae*	4	0.3	0.2	0 – 0.6	Poly	
*Agrodiaetus alcestis*	6	0.8	0.4	0 – 1.5	Poly	Poly
*Agrodiaetus altivagans*	9	1.8	1.5	0 – 5.5	Poly	
*Agrodiaetus antidolus*	4	0.3	0.3	0 – 0.7	Poly	Poly
*Agrodiaetus arasbarani*	2	1.0		---	Poly	Poly
*Agrodiaetus baytopi*	4	1.9	1.2	0.5 – 3.1	Poly	Poly
*Agrodiaetus birunii*	10	0.2	0.2	0 – 0.7	Para	Para
*Agrodiaetus caeruleus*	3	0.5	0.5	0 – 1	Mono	Mono
*Agrodiaetus carmon*	4	1.3	0.6	0.6 – 2	Poly	Poly
*Agrodiaetus cyaneus*	6	0.2	0.2	0 – 0.7	Poly	Poly
*Agrodiaetus damocles*	4	1.1	0.8	0.1 – 1.8	Poly	Poly
*Agrodiaetus damon*	5	1.6	0.8	0 – 2.9	Mono	Mono
*Agrodiaetus damone*	3	0.6	0.0	0.6 – 0.6	Para	Para
*Agrodiaetus dantchenkoi*	6	0.0	0.0	0 – 0	Poly	Poly
*Agrodiaetus darius*	3	0.0	0.0	0 – 0	Mono	Mono
*Agrodiaetus demavendi*	17	2.1	1.3	0 – 3.6	Poly	Poly
*Agrodiaetus elbursicus*	9	0.5	0.8	0 – 2.1	Poly	Poly
*Agrodiaetus erschoffii*	3	0.2	0.2	0 – 0.3	Mono	Mono
*Agrodiaetus fabressei*	3	0.1	0.1	0 – 0.2	Poly	Poly
*Agrodiaetus femininoides*	2	1.8		---	Poly	Poly
*Agrodiaetus firdussii*	9	0.5	0.4	0 – 1.3	Poly	Mono
*Agrodiaetus fulgens*	2	0.2		---	Poly	Poly
*Agrodiaetus glaucias*	2	0.2		---	Mono	Mono
*Agrodiaetus gorbunovi*	5	0.1	0.1	0 – 0.2	Para	
*Agrodiaetus haigi*	3	0.0	0.0	0 – 0	Poly	
*Agrodiaetus hamadanensis*	4	0.4	0.3	0 – 0.7	Mono	Mono
*Agrodiaetus hopfferi*	3	1.5	1.3	0.2 – 2.8	Para	Para
*Agrodiaetus huberti*	7	0.5	0.4	0 – 1.3	Poly	
*Agrodiaetus humedasae*	2	0.2		---	Mono	Mono
*Agrodiaetus iphidamon*	4	0.0	0.0	0 – 0	Mono	Mono
*Agrodiaetus iphigenia*	8	0.7	0.6	0 – 2	Mono	Mono
*Agrodiaetus iphigenides*	3	1.6	0.8	0.7 – 2.2	Poly	Poly
*Agrodiaetus kanduli*	2	2.7		---	Poly	
*Agrodiaetus kendevani*	2	5.4		---	Poly	
*Agrodiaetus khorasanensis*	2	0.5		---	Mono	
*Agrodiaetus klausschuriani*	3	0.0	0.0	0 – 0	Mono	Mono
*Agrodiaetus kurdistanicus*	3	0.0	0.0	0 – 0	Poly	Poly
*Agrodiaetus lorestanus*	2	0.0		---	Mono	
*Agrodiaetus lycius*	2	0.8		---	Mono	Mono
*Agrodiaetus menalcas*	5	0.6	0.4	0 – 1.3	Mono	Mono
*Agrodiaetus merhaba*	3	2.4	1.2	1.1 – 3.5	Poly	Poly
*Agrodiaetus mithridates*	2	4.6		---	Poly	Poly
*Agrodiaetus mofidii*	2	1.0		---	Poly	Poly
*Agrodiaetus nephohiptamenos*	2	0.0		---	Mono	
*Agrodiaetus ninae*	5	0.7	0.3	0.2 – 1.3	Poly	Poly
*Agrodiaetus paulae*	2	0.0		---	Para	Para
*Agrodiaetus phyllides*	4	0.7	0.2	0.4 – 0.9	Poly	Poly
*Agrodiaetus phyllis*	4	1.7	0.7	0.5 – 2.5	Para	Mono
*Agrodiaetus pierceae*	3	0.3	0.2	0.2 – 0.5	Mono	Mono
*Agrodiaetus poseidon*	5	0.5	0.4	0 – 1	Poly	Mono
*Agrodiaetus posthumus*	3	0.1	0.1	0 – 0.2	Mono	Mono
*Agrodiaetus pseudactis*	2	1.0		---	Poly	
*Agrodiaetus pseudoxerxes*	2	1.8		---	Poly	Poly
*Agrodiaetus putnami*	3	0.0	0.0	0 – 0	Poly	
*Agrodiaetus ripartii*	17	1.4	0.8	0 – 3.3	Poly	Poly
*Agrodiaetus rjabovi*	2	1.1		---	Mono	Mono
*Agrodiaetus rovshani*	4	0.2	0.2	0 – 0.4	Mono	Mono
*Agrodiaetus sekercioglu*	2	0.5		---	Poly	
*Agrodiaetus shahrami*	2	0.3		---	Poly	Poly
*Agrodiaetus sigberti*	2	0.9		---	Poly	
*Agrodiaetus surakovi*	2	0.2		---	Para	Poly
*Agrodiaetus tankeri*	3	1.7	0.7	1 – 2.3	Poly	Poly
*Agrodiaetus tenhageni*	2	0.0		---	Mono	Mono
*Agrodiaetus turcicolus*	5	0.8	0.4	0 – 1.3	Poly	
*Agrodiaetus turcicus*	4	0.8	0.3	0.5 – 1.1	Mono	Mono
*Agrodiaetus valiabadi*	2	0.0		---	Mono	Mono
*Agrodiaetus vanensis*	5	0.5	0.3	0 – 0.8	Mono	
*Agrodiaetus wagneri*	2	0.1		---	Para	
*Agrodiaetus zapvadi*	4	0.0	0.1	0 – 0.1	Poly	
*Agrodiaetus zarathustra*	2	0.0		---	Mono	Mono
*Arhopala achelous*	6	1.3	0.9	0.2 – 3.1	Poly	Poly
*Arhopala antimuta*	2	1.1		---	Mono	Mono
*Arhopala atosia*	3	2.9	1.7	1 – 4.3	Poly	Poly
*Arhopala barami*	2	0.3		---	Mono	Mono
*Arhopala democritus*	2	1.0		---	Mono	Mono
*Arhopala epimuta*	6	0.2	0.3	0 – 0.8	Para	Para
*Arhopala labuana*	2	0.5		---	Mono	Mono
*Arhopala major*	2	0.3		---	Mono	Mono
*Arhopala moolaiana*	2	2.3		---	Mono	Mono
*Aricia agestis*	6	0.7	1.0	0 – 2.4	Poly	Poly
*Aricia artaxerxes*	3	1.8	0.5	1.2 – 2.1	Poly	
*Celastrina argiolus*	2	1.3		---	Mono	Mono
*Chrysoritis nigricans*	2	1.1		---	Mono	Mono
*Chrysoritis pyroeis*	2	0.0		---	Mono	Mono
*Cyaniris semiargus*	4	1.0	0.4	0.3 – 1.5	Mono	Mono
*Favonius cognatus*	3	0.3	0.2	0.1 – 0.5	Poly	Poly
*Favonius jezoensis*	2	0.6		---	Mono	Mono
*Favonius korshunovi*	2	0.4		---	Mono	Mono
*Favonius orientalis*	3	0.7	0.5	0.1 – 1	Poly	Mono
*Favonius saphirinus*	8	1.1	0.7	0 – 2	Mono	Mono
*Favonius taxila*	3	0.1	0.1	0 – 0.1	Mono	Mono
*Favonius ultramarinus*	5	1.0	0.4	0.4 – 1.4	Poly	Poly
*Favonius yuasai*	3	0.5	0.4	0.1 – 0.8	Mono	Mono
*Flos anniella*	2	2.6		---	Mono	Mono
*Jalmenus evagoras*	12	0.6	0.3	0.2 – 1.1	Mono	Mono
*Lampides boeticus*	4	4.3	4.5	0.2 – 8.7	Poly	Mono
*Lucia limbaria*	2	1.7		---	Mono	Mono
*Lycaeides melissa*	5	0.7	0.5	0 – 1.2	Poly	Poly
*Lycaena alciphron*	2	2.7		---	Mono	Mono
*Lysandra albicans*	3	0.9	0.1	0.8 – 0.9	Para	
*Lysandra bellargus*	6	0.2	0.3	0 – 0.8	Mono	Mono
*Lysandra coridon*	5	1.6	0.5	0.7 – 2.1	Poly	Poly
*Lysandra corydonius*	4	1.7	1.2	0 – 2.7	Poly	Poly
*Lysandra ossmar*	2	2.1		---	Poly	
*Maculinea alcon*	7	0.0	0.1	0 – 0.2	Poly	Mono
*Maculinea arion*	10	0.2	0.2	0 – 0.6	Para	Para
*Maculinea arionides*	4	0.5	0.4	0 – 0.9	Poly	Poly
*Maculinea nausithous*	3	2.2	0.3	1.9 – 2.4	Mono	Mono
*Maculinea rebeli*	3	0.1	0.2	0 – 0.3	Poly	
*Maculinea teleius*	5	0.9	0.5	0.2 – 1.6	Mono	Mono
*Meleageria daphnis*	4	2.1	0.4	1.5 – 2.6	Poly	Mono
*Meleageria marcida*	2	4.4		---	Poly	
*Neolysandra fatima*	2	0.0		---	Mono	Mono
*Neozephyrus japonicus*	2	4.8		---	Poly	
*Plebejus argus*	5	1.0	0.8	0 – 1.9	Mono	Mono
*Polyommatus amandus*	3	2.6	2.0	0.3 – 3.8	Para	Para
*Polyommatus cornelia*	3	1.1	0.5	0.6 – 1.5	Para	Para
*Polyommatus dorylas*	4	1.6	0.4	1.2 – 2.3	Mono	Mono
*Polyommatus eroides*	2	1.4		---	Poly	Poly
*Polyommatus escheri*	2	2.0		---	Mono	Mono
*Polyommatus icarus*	8	2.2	2.3	0 – 6.8	Poly	Poly
*Polyommatus menelaos*	2	0.0		---	Mono	Mono
*Polyommatus myrrhinus*	3	0.1	0.1	0 – 0.1	Mono	Mono
*Polyommatus thersites*	5	0.9	0.6	0 – 1.6	Mono	Mono
*Pseudophilotes vicrama*	2	0.0		---	Mono	Mono
*Quercusia quercus*	2	0.6		---	Mono	Mono
*Vacciniina alcedo*	2	0.0		---	Mono	Mono

Mean and range of intraspecific nucleotide divergences for 133 Lycaenidae species, using Kimura's two parameter model. The column "Monophyly" states if conspecific sequences form a monophylum ("Mono"), a paraphylum ("Para") or a polyphylum ("Poly") and the subsequent column gives the corrected status (if presumable errors are excluded and critical taxa are lumped together).

### Interspecific divergence

The average divergence in 236348 interspecific comparisons is 9.38% (SE = 3.65%) ranging from 0.0% to 23.2% (between *Baliochila minima *and *Agrodiaetus poseidon*). Of these, 57562 are congeneric comparisons with an average divergence of 5.07% (SE = 1.73%) ranging from 0.0% (between 23 *Agrodiaetus *as well as 3 *Maculinea *species pairs) to 12.4% (between *Arhopala abseus *and *Arhopala ace*). 94% of those comparisons are within *Agrodiaetus*. Only congeneric comparisons were included in subsequent analyses in order to make comparisons feasible across taxonomic levels. Table 2 lists mean interspecific divergences in genera of which at least two species are represented in the data set. Sequence divergence in 95% of interspecific (congeneric) comparisons is above 1.9%, and 87.6% of such comparisons reveal distances above 3%.

**Table 2:** Interspecific nucleotide divergences

**Genus**	**No. of species**	**Mean percent divergence**	**Standard error (%)**	**Range (%)**
*Acrodipsas*	9	3.1	1.0	0.5 – 5.7
*Agriades*	2	4.7		---
*Agrodiaetus*	117	5.1	1.7	0 – 10.1
*Arhopala*	30	6.8	1.7	0.4 – 12.4
*Aricia*	7	3.4	1.9	0.2 – 7.5
*Chrysoritis*	19	7.0	2.5	0.8 – 10.9
*Euphilotes*	2	10.3		---
*Favonius*	9	4.0	0.9	0.1 – 5.4
*Glaucopsyche*	2	1.3		---
*Lycaeides*	3	1.7	0.9	0.5 – 3.0
*Lycaena*	9	4.5	1.1	1.2 – 6.8
*Lysandra*	9	2.2	0.7	0.7 – 4.0
*Maculinea*	7	2.8	1.4	0 – 6.0
*Meleageria*	2	2.6	1.6	0.1 – 4.4
*Neolysandra*	5	4.6	1.6	1 – 6.3
*Phengaris*	3	3.8	2.1	1.3 – 5.1
*Plebejus*	5	5.6	1.6	2.4 – 7.4
*Polyommatus*	12	5.9	2.5	0.1 – 10.5
*Pseudophilotes*	4	2.7	1.6	0.6 – 4.5
*Satyrium*	3	4.5	0.5	4 – 4.9
*Trimenia*	2	6.1		---
*Turanana*	2	4.8		---
*Vacciniina*	3	7.2	0.3	6.8 – 7.5

Mean and range of interspecific nucleotide divergences for species in 22 Lycaenidae genera, using Kimura's two parameter model

### The barcode gap

**Figure 1:**
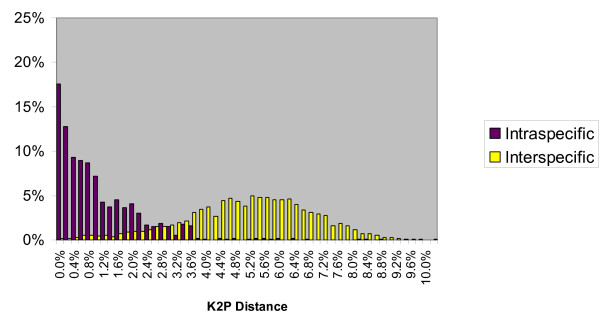
**Frequency distribution of intraspecific and interspecific (congeneric) genetic divergence in Lycaenidae**. Total number of comparisons: 1189 intraspecific and 57562 interspecific pairs across 315 Lycaenidae species. Divergences were calculated using Kimura's two parameter (K2P) model.

**Figure 2:**
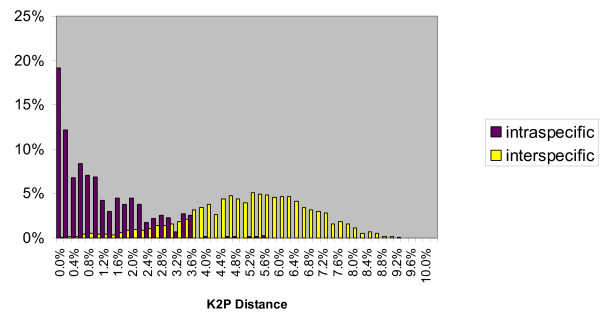
**Frequency distribution of intraspecific and interspecific (congeneric) genetic divergences in *Agrodiaetus***. Total number of comparisons: 737 intraspecific and 54209 interspecific pairs across 114 *Agrodiaetus *species. Divergences were calculated using Kimura's two parameter (K2P) model.

As apparent in Figure 1 (and Figure 2 for comparisons within *Agrodiaetus *only) no gap exists between intraspecific and interspecific divergences. Since some (0.14%) interspecific divergences are as low as 0% no safe threshold can be set to strictly avoid false negatives. Although species pairs with such low divergences include some whose taxonomic status as distinct species is debatable, they also include many pairs which are well differentiated in phenotype, have a very different karyotype (in *Agrodiaetus*), and occur sympatrically without any evidence for interbreeding. Examples include *Agrodiaetus peilei – A. morgani *(0.0%), *Agrodiaetus fabressei – A. ainsae *(0.2%), *Agrodiaetus peilei – A. karindus *(0.2%), *Polyommatus myrrhinus – P. cornelia *(0.4%), or *Agrodiaetus poseidon – A. hopfferi *(0.6%).

**Figure 3:**
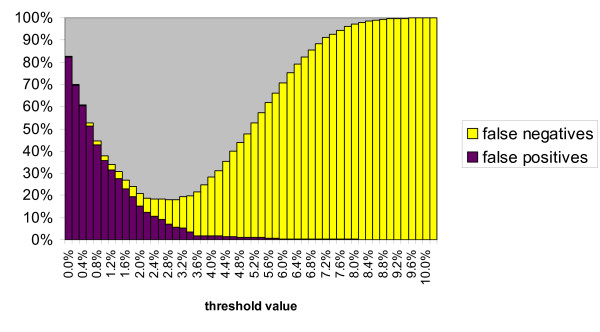
**Cumulative error based on false positives plus false negatives for each threshold value in 315 Lycaenidae species including only congeneric comparisons**. The optimum threshold value is 2.8%, where error is minimized at 18.0%.

**Figure 4:**
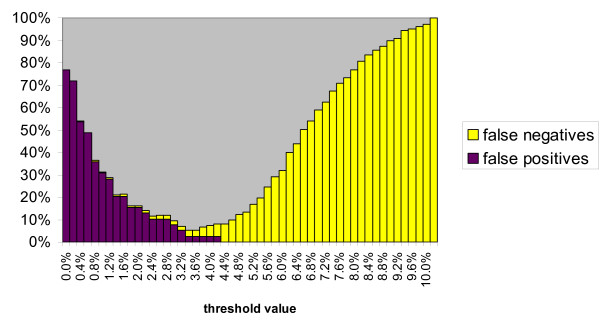
**Cumulative error based on false positives plus false negatives for each threshold value in 30 *Arhopala *species**. The optimum threshold value is 3.4%, where error is minimized at 5.3%.

The minimum cumulative error based on false positives plus false negatives is 18% at a threshold level of 2.8% (Figure 3). Minimum errors are very similar for *Agrodiaetus *(18.6% at 3.0% threshold, not shown) and other Lycaenidae (18.6% at 2.0% threshold, not shown), but much lower in *Arhopala *(5.3% at 3.4% threshold, Figure 4).

**Figure 5:**
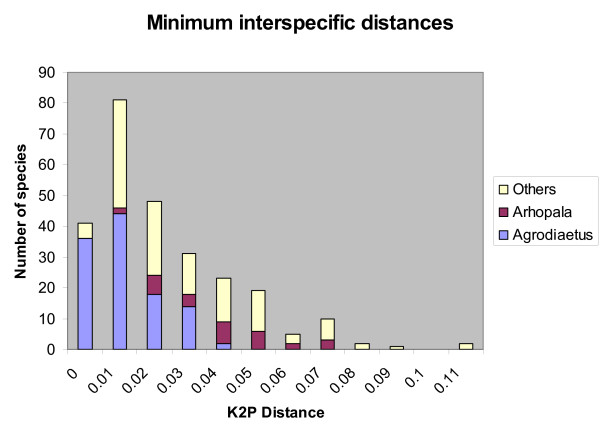
Frequency distribution of minimum interspecific (congeneric) genetic distances across 263 Lycaenidae species.

For safe identification, minimum distances between species (Figure 5) are critical and not average distances. In *Agrodiaetus*, all but two species (= 98.3%) have close relatives with interspecific distances below 3%. In the other genera combined, "only" 74% of taxa are affected but this lower rate is probably due to undersampling and would rise, if more sequences of more closely related species become available for the analysis.

### Identification with NJ tree profile

The approach of species identification with a Neighbour-Joining (NJ) tree profile as proposed by [9] does not necessarily depend on the barcoding gap but on the coalescence of conspecific populations and the monophyly of species (details see **Data analysis**).

The success rate in the identification of our Lycaenidae data set with this method was 58%. Five out of 158 misidentifications or ambiguous identifications (3.2%) can be attributed to incorrectly identified specimens (*Lampides boeticus, Neozephyrus japonicus, Agrodiaetus kendevani*, see above). Further 90 cases (57%) were among closely related sister species whose taxonomic status is in dispute (Table 3). If these cases are not taken into account (i.e. counted as successful identifications, an unrealistic best case scenario for barcoding success), the success rate would rise to 84%. In *Agrodiaetus *the success rate would remain lower (79%) while in the remaining genera it would reach 91%. But even with these corrections, 61 cases of misidentifications (16%) remain, 46 of these in *Agrodiaetus *(affected taxa in Table 4). The complete Neighbour-joining tree (available for download as additional file 1: NJ-tree) shows the reason for this failure: Only 46% of conspecific sequences form a monophyletic group on this tree while the others are either paraphyletic (10%) or even polyphyletic (44%). In *Agrodiaetus*, only 34% of species are monophyletic (Table 1), while the others are paraphyletic (11%) or polyphyletic (55%). If incorrectly identified specimens are excluded and critical taxa (Table 3) are lumped together, still only 59% of species are monophyletic (43% in *Agrodiaetus*) while 7% are paraphyletic and 34% polyphyletic (49% in *Agrodiaetus*).

**Table 3:** Sister species or species complexes with disputable species borders

*Agrodiaetus altivagans/****damocles***/*ectabanensis/gorbunovi/kanduli/maraschi/wagneri *[30, 41]
*Agrodiaetus artvinensis/bilgini/****firdussii***/*pseudactis/sigberti *[30]
*Agrodiaetus aserbeidschanus/huberti/****ninae***/*turcicolus/zapvadi *[30]
*Agrodiaetus ****baytopi***/*iphicarmon *[30]
*Agrodiaetus ****carmon***/*schuriani *[30]
*Agrodiaetus ****cyaneus***/*kermansis/paracyaneus*
*Agrodiaetus ****demavendi***/*lorestanus *[30]
*Agrodiaetus khorasanensis/nephohiptamenos/****ripartii ***[30]
*Agrodiaetus ****phyllis***/*vanensis *[30]
*Agrodiaetus ****poseidon***/*putnami *[30]
*Agrodiaetus sekercioglu/****surakovi ***[30]
*Aricia ****agestis***/*artaxerxes *[30]
*Lysandra albicans/caelestissimus/****coridon***/*gennargenti *[30]
*Lysandra caucasicus/****corydonius***/*ossmar *[30]
*Maculinea ****alcon***/*rebeli *[30, 54]
*Meleageria ****daphnis***/*marcida *[30, 42, 54]
*Polyommatus andronicus/****icarus ***[30]
*Polyommatus ****eros***/*eroides *[30]

List of disputable species complexes due to e.g. incomplete speciation and gene flow or, in *Agrodiaetus*, very similar phenotype and only slight differences in karyotype. The taxonomically oldest name is marked in bold.

**Table 4:** Taxa misidentified with the NJ tree profile approach

*Agrodiaetus ****admetus ***(78–80)/***demavendi ***(≈67)/*nephohiptamenos *(≈90)
*Agrodiaetus ****ainsae ***(108–110)/***fabressei ***(90)
*Agrodiaetus ****alcestis ***(19–21)/***dantchenkoi ***(40–42)/*eriwanensis *(28–32)/*interjectus *(29–32)
*Agrodiaetus ****altivagans ***(18–22)/*ciscaucasicus *(16)
*Agrodiaetus ****antidolus ***(42)/***femininoides ***(27)/*kurdistanicus *(62)
*Agrodiaetus ****arasbarani ***(25)/*elbursicus *(16)/*lukhtanovi *(22)/***paulae ***(17)/*zarathustra *(≈22)
*Agrodiaetus baytopi *(27–28)/***tankeri ***(20–21)
*Agrodiaetus ****birunii ***(10–11)/*brandti *(19)
*Agrodiaetus ****carmon ***(81–82)/*surakovi *(50)
*Agrodiaetus ciscaucasicus *(16)/***mofidii ***(35)
*Agrodiaetus cyaneus *(19)/***pseudoxerxes ***(15–16)
*Agrodiaetus damone *(66–68)/***iphigenides ***(67)*juldusus *(67)/*karatavicus *(67)/***phyllides ***(67)
*Agrodiaetus ****elbursicus ***(17)/*turcicolus *(20)
*Agrodiaetus hopfferi *(15)/***poseidon ***(19–22)
*Agrodiaetus lorestanus *(68)/***ripartii ***(90)
*Arhopala ****achelous***/*muta*
*Favonius ****cognatus***/***ultramarinus***
*Maculinea ****arion***/***arionides***
*Polyommatus ****amandus abdelaziz ***/*Meleageria daphnis*
*Polyommatus ****cornelia***/*myrrhinus*

List of taxa which were misidentified with the NJ tree profile approach (excluding possible errors and critical taxa listed in Tab.3). Misidentified test taxa (in bold font) and their identifications are placed jointly in a single line. Haploid chromosome numbers of *Agrodiaetus *species (taken from [29, 30, 44]) are given in parenthesis.

## Conclusion

We found an upper limit for intraspecific sequence divergences in a wide range of species of the diverse butterfly family Lycaenidae, but no lower limit for interspecific divergences and thus no barcoding gap. This result is especially well documented in the comprehensively sampled genus *Agrodiaetus *(114 of ca 130 recognized species sequenced) while the smaller overlap in *Arhopala *can be attributed to the lower percentage of species sampled (33 of more than 200 species). The choice of species by [46] was to maximize coverage of divergent clades while minimizing the total number of species which is a common and sensible approach for phylogenetic studies, but undermines the power of such sequence data as critical tests for the barcoding approach. The general level of sequence divergence is not exceptionally low in Lycaenidae compared to other Lepidoptera. The mean congeneric interspecific sequence divergence of 5.1% in Lycaenidae (5.1% in *Agrodiaetus *and 5.0% in the other genera) was only slightly lower than the mean value of 6.6% found by [2] in various families of Lepidoptera.

We thus confirm the results of Meyer & Paulay [17] and Meier et al. [18]. Our results also agree with those from a recent study in the Neotropical butterfly subfamily Ithomiinae (Nymphalidae) [47] which records highly variable levels of divergence in mtDNA (*COI *&*COII*) between taxa of the same rank. Our results however fail to agree with those of Barrett & Hebert [9] on arachnids. In that study the mean percent sequence divergence between congeneric species was 16.4% (SE = 0.13) and thus three times higher than in our study while the divergence among conspecific individuals was only slightly higher with 1.4% (SE = 0.16). The contradiction between our study and theirs can be explained by the very incomplete and sparse taxon sampling in their data set amounting to just 1% of the species contained within the families. We conclude that the reported existence of a barcode gap in arachnids appears to be an artifact based on insufficient sampling across taxa.

Despite these difficulties, species identification of unidentified samples with the help of barcodes is entirely possible. The NJ tree profile approach which does not rely on a barcode gap enabled the correct assignment of many sequences, and other methods (e.g. applying population genetic approaches) might further increase the success rate. However, 17% of test sequences could still not be identified correctly, even in some sympatric species pairs which clearly differ in phenotype and chromosome number (e.g. *Agrodiaetus ainsae *[n = 108–110]/*fabressei *[n = 90], *Agrodiaetus hopfferi *[n = 15]/*poseidon *[n = 19–22]). The main reason for this failure is that a large proportion of species are not reciprocally monophyletic, e.g. due to incomplete lineage sorting, which is in accordance with a previous study [48]. Moreover, the success with this method is again completely dependent on comprehensive sampling. If the correct species is not included in the profile, the assignment must by necessity be incorrect and misleading. Because of the non-existence of a barcoding gap, this error will often be impossible to detect. This limits possible applications of the barcoding approach. For example, cryptic species can only be detected with the help of a barcoding approach at high genetic divergence from all phenotypically similar species. An example is *Agrodiaetus paulae *which was discovered in this way [41]. In contrast, and on the one hand, the sympatric species pairs *Agrodiaetus ainsae-fabressei, A. hopfferi-poseidon *and *A. morgani-peilei *would have gone unnoticed by barcoding approaches even though their strong phenotypical and karyological differentiation (n = 108 vs. n = 90, n = 15 vs. n = 19–22 and n = 27 vs. n = 39, respectively) clearly indicates their specific distinctness. On the other hand, sequence divergence in what is currently believed to represent one species does not *per se *prove the specific distinctness of the entities in question. In *Polyommatus icarus *or *P. amandus*, for example, the high divergences between North African and Eurasiatic samples is a strong hint for the presence of unrecognized cryptic species, but this needs to be rigorously tested with sequence data from samples that cover the geographic range more comprehensively. Also in practical application the problem of misidentified specimens and sequences in GenBank remains a real threat to the accuracy of barcode-based identifications. An example is the GenBank sequence AB192475 of *Lampides boeticus *which is also used in the CBOL database (see above). This underscores the importance of voucher specimens and documentation of locality data, an issue raised by barcoding supporters but unfortunately still much neglected by GenBank. Another case of misidentification (GenBank sequence AF170864 of *Plebejus acmon *which was originally submitted as *Euphilotes bernardino*) [30] has already been corrected with the help of the voucher specimen.

In conclusion, the barcoding approach can be very helpful, e.g. in identifying early stages of insects or when only fragments of individuals are available for analysis. However, correct identification requires that all eligible species can be included in the profile and that sufficient information is available on the amount of intraspecific genetic variation and genetic distance to closely related species.

The barcoding procedure is not very well suited for identifying species boundaries but it may help to give minimum estimates of species numbers in very diverse and inadequately known taxonomic groups at single localities. Our case study on *Agrodiaetus *shows that a substantial number of species would have gone unnoticed by the barcoding approach as 'false negatives'. Thus, especially in clades where many species have evolved rapidly as a result of massive radiations with minimum sequence divergence, the barcoding approach holds little promise of meeting the challenge of rapid and reliable identification of large samples. Yet, it is exactly these situations which pose the most problematic tasks in the morphological identification of insects.

Although molecular data can be helpful in discovering new species, a large genetic divergence is not sufficient proof since it must be corroborated by other data. Furthermore, most closely related species which are difficult to identify with traditional means, are also similar genetically and would go unnoticed by an isolated barcoding approach. Mathematical simulations have shown that populations have to be isolated for more than 4 million generations (i.e. 4 million years in the mostly univoltine *Agrodiaetus *species) for two thresholds proposed by the barcoding initiative (reciprocal monophyly, and a genetic divergence between species which is 10 times greater than within species) to achieve error rates less than 10% [49]. This might help to explain why the barcoding approach appears to be more successful in the Oriental genus *Arhopala *which is thought to represent a phylogenetically older lineage of Lycaenidae estimated to be about 7–11 Million years old [50], while the origin of the Palaearctic genus *Agrodiaetus *is dated at only 2.5–3.8 Million years [44].

Our data show that the lack of a barcoding gap and reciprocal monophyly in Lycaenidae is not confined to the genus *Agrodiaetus *with its extraordinary interspecific variation in chromosome numbers, but also to other genera of Lycaenidae with stable chromosome numbers. It should also be noted that in *Agrodiaetus *there is neither evidence for exceptional rapid radiation as in cichlids of the East African lakes [51] nor for unusual (i.e. sympatric) speciation patterns caused by karyotype evolution. Rather, karyotype diversification seems to have been a mere by-product of the usual mode of allopatric speciation [29,30,44].

## Methods

### Data sources

**Figure 6:**
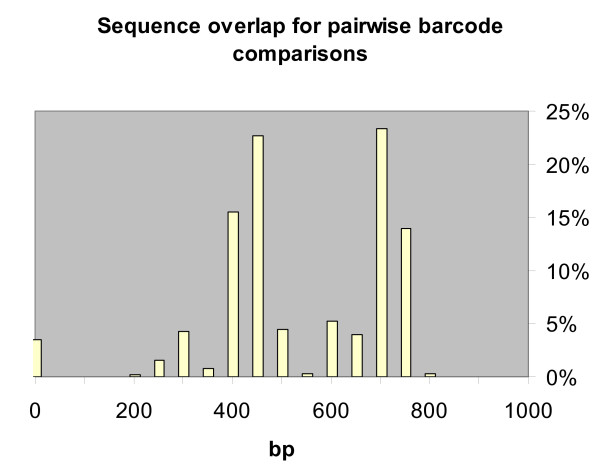
**Sequence overlap for pairwise barcode comparisons**. Length of sequence overlap in 246229 cross-comparisons of 694 aligned sequences

A total of 694 barcode sequences were used for our analysis. We used a 690 bp fragment at the 5' end of cytochrome c oxidase subunit I (*COI*) of 309 Lycaenidae sequences from a molecular phylogenetic study by Wiemers [30]. Most sequences belong to *Agrodiaetus *(198), the others (111) mostly to closely related Polyommatinae. All sequences have been deposited in GenBank [52] (AY556844-AY556867, AY556869-AY556963, AY556965-AY557155) with LinkOuts provided to images of the voucher specimens deposited with MorphBank [53]. These sequences were supplemented by 385 further sequences of Lycaenidae deposited in GenBank as of March, 2006 (Table 5). They include sequences from further studies on *Agrodiaetus *[29,44], the Palaearctic genus *Maculinea *[54], Nearctic *Lycaeides melissa *[55], the Oriental genus *Arhopala *[46,50], the Australian genera *Acrodipsas *[56] and *Jalmenus *[57], and the South African *Chrysoritis *[58] as well as a few sequences which have only been used as outgroups in non-Lycaenidae studies (e.g. [59,60]). Sequence length in the 5' region as defined by CBOL ranged between 240 bp and the maximum of 987 bp. (18 *COI *sequences from a study on *Japonica *only contained a 3'end fragment and therefore were not included.) Of these, 89% are at least 648 bp long as recommended by CBOL and 98% at least 500 bp long which is deemed sufficient for barcode sequences [13]. However, sequence overlap for sequences from different studies was sometimes lower because of slightly different sequence locations within the barcode region (Figure 6). It should be noted that these inconsistencies in barcode comparisons are a common situation in barcode sequences due to differences in primer use (e.g. [2]).

**Table 5:** Material

**GenBank accession no.**	**Number of sequences**	**Reference**	***Taxa in focus***
AY556844 – AY556867 AY556869 – AY556963 AY556965 – AY557155	309	[30, 41]	*Agrodiaetus*
AY496709 – AY496821 AY502111 – AY502112 AY953984 – AY954025	157	[29, 44]	*Agrodiaetus*
AY235861 – AY235903 AY235955 – AY236006	52	[46, 50]	*Arhopala*
AY675402 – AY675448	47	[54]	*Maculinea*
DQ234691 – DQ234695	5	[55]	*Lycaeides*
AY091712 – AY091741	30	[56]	*Acrodipsas*
DQ249942 – DQ249953	12	[57]	*Jalmenus*
AF279217 – AF279244	28	[58]	*Chrysoritis*
AF170864	1	[59]	Papilionidae
AY350456 – AY350459	4	[60]	Lepidoptera
DQ018938 – DQ018948	11	[67]	Papilionoidea & Hesperioidea
AB195510 – AB195545	36	Odagiri *et al*. (unpubl.)	*Favonius*
AB192475 – AB192476	2	Tanikawa *et al*. (unpubl.)	Hesperiidae

List of GenBank accession numbers used for analysis including references and taxa which were the focus of these studies

### Laboratory protocols

DNA was extracted from thorax tissue recently collected and preserved in 100% ethanol using Qiagen^® ^DNeasy Tissue Kit according to the manufacturer's protocol for mouse tail tissue. In a few cases only dried material was available and either thorax or legs were used for DNA extraction.

Amplification of DNA was conducted using the polymerase chain reaction (PCR). The reaction mixture (for a total reaction volume of 25 μl) included: 1 μl DNA, 16.8 μl ddH20, 2.5 μl 10 × PCR II buffer, 3.2 μl 25 mM MgCl2, 0.5 μl 2 mM dNTP-Mix, 0.25 μl Taq Polymerase and 0.375 μl 20 pm of each primer. The two primers used were: 

Primer 1:  k698  TY-J-1460  TAC AAT TTA TCG CCT AAA CTT CAG CC   [61]

Primer 2:  Nancy  C1-N-2192  (CCC) GGT AAA ATT AAA ATA TAA ACT TC  [61]

PCR was conducted on thermal cyclers from Biometra^® ^(models Uno II or T-Gradient) or ABI Biosystems^® ^(model GeneAmp^® ^PCR-System 2700) using the following profiles:

Initial 4 minutes denaturation at 94°C and 35 cycles of 30 seconds denaturation at 94°C, 30 seconds annealing at 55°C and 1 minute extension at 72°C.

PCR products were purified using purification kits from Promega^® ^or Sigma^® ^and checked with agarose gel electrophoresis before and after purification.

Cycle sequencing was carried out on Biometra^® ^T-Gradient or ABI Biosystems^® ^GeneAmp^® ^PCR-System 2700 thermal cyclers using sequencing kits of MWG Biotech^® ^(for Li-cor^® ^automated sequencer) or ABI Biosystems^® ^(for ABI^® ^377 automated sequencer) according to the manufacturers' protocols and with the following cycling times: initial 2 minutes denaturation at 95°C and 35 cycles of 15 seconds denaturation at 95°C, 15 seconds annealing at 49°C and 15 seconds extension at 70°C. Primers used were the same as for the PCR reactions for the ABI (primer 1 was used for forward and primer 2 for independent reverse sequencing), but for Li-cor truncated and labelled primers were used with 3 bases cut off at the 5' end and labelled with IRD-800. For ABI sequencing the products were cleaned using an ethanol precipitation protocol. Electrophoresis of sequencing reaction products was carried out on Li-cor^® ^or ABI^® ^377 automated sequencers using the manufacturer's protocols.

### Data analysis

Sequences were aligned with BioEdit 7.0.4.1 [62] and pruned to a maximum of 987 bp, the section proposed by CBOL for barcoding. Pairwise sequence divergences were calculated separately for intraspecific as well as for interspecific, but intrageneric comparisons with Mega 3.1 [63] using Kimura's two parameter (K2P) distance model. This is not necessarily the best model to analyze the data (see [64]), but it was chosen to facilitate comparisons with other barcode studies of Hebert and co-workers [1,9-12,16] who have been using this model. Distance tables were processed to calculate divergence means (incl. standard errors and ranges) within and between species.

The taxonomy was taken from GenBank in most cases but two minor spelling inconsistencies were corrected. In four cases where a taxon within *Agrodiaetus *was treated as a species taxon by one author but only as a subspecies by another, we matched them by treating those taxa as distinct species. The generic subdivision of Lycaenidae is very much in flux. Some genera are only treated as subgenera by some authors and many genera (like *Polyommatus *or *Plebejus*) are probably paraphyletic or polyphyletic, however we undertook no revision of the GenBank taxonomy since it appeared consistent enough for our analysis. The remaining inconsistencies only affect few taxa in our analysis and include the treatment of *Sublysandra *(distinct genus or subgenus of *Polyommatus*), *Eumedonia *(distinct genus or subgenus of *Aricia*), *Otnjukovia *(synonym to *Turanana*), *Maculinea *(synonym to *Phengaris*) and *Callipsyche *(synonym to *Satyrium*). (A complete list of sequences with corresponding taxa names and voucher numbers is found in the additional file 1: NJ tree.)

A Lycaenidae species profile was created according to [9]. Of the 694 barcode sequences, we excluded 9 short *Arhopala *sequences with a barcode length of only 240 bp. (To check the position of those sequences, a separate analysis was run containing only the *Arhopala *sequences.) Of the remaining 685 sequences, we randomly selected 1 sequence from each of the 308 Lycaenidae species for inclusion into a *COI *species profile. We chose a sequence of *Apodemia mormo *(GenBank accession number AF170863) from the family Riodinidae as outgroup because this family appears to represent the sister group to Lycaenidae [65-67]. The other 377 sequences which had not been included in the profile were used as "test" sequences: They were singly added to the test profile in repeated Neighbour-joining analyses and their "classification success" was recorded. A test was recorded as successful if the test sequence grouped most closely with the conspecific profile sequence and not with another species. Results of three GenBank sequences which were not identified to species level (all belonging to the genus *Agrodiaetus*) were not counted. After the classification test, another NJ analysis was run including all sequences in order to understand possible failures in classification. The main reason for using the Neighbour-joining as a tree-building method is its computational efficiency. Although this method is well suited for grouping closely related sequences, it should be noted that other methods (such as Maximum Parsimony, Maximum Likelihood or Bayesian inference of phylogeny) are usually superior in constructing phylogenetic trees.

## Competing interests

The author(s) declare that they have no competing interests.

## Authors' contributions

MW carried out the molecular genetic studies, sequence alignment, statistical analysis and drafted the manuscript. KF participated in the design of the study and the statistical analysis and helped to draft the manuscript. All authors read and approved the final manuscript.

## Supplementary Material

Additional file 1 Neighbour-joining tree (Distance model: Kimura-2-Parameter) of profile and test taxa; includes a list of GenBank sequences with taxa names and corresponding voucher codes. Click here for file
